# Revealing the Roles of the SH3GLB1-Hydrogen Peroxide Axis in Glioblastoma Multiforme Cells

**DOI:** 10.32604/or.2025.071258

**Published:** 2026-01-19

**Authors:** Wei-Ting Hsueh, Kwang-Yu Chang, Chin-Chuan Tsai, Kuan-Tso Chen, Kuen-Jang Tsai, Zi-Xuan Hong, Chan-Chuan Liu, Jui-Mei Chu, Li-Ying Qiu, Yu-Yan Lan, Chia-Hung Chien

**Affiliations:** 1 Department of Oncology, National Cheng Kung University Hospital, College of Medicine, National Cheng Kung University, Tainan, 70101, Taiwan; 2 National Institute of Cancer Research, National Health Research Institutes, Tainan, 70456, Taiwan; 3 Department of Pharmacology, College of Medicine, National Cheng Kung University, Tainan, 70101, Taiwan; 4 Department of Chinese Medicine, E-Da Dachang Hospital, Kaohsiung, 80706, Taiwan; 5 The School of Chinese Medicine for Post-Baccalaureate, I-Shou University, Kaohsiung, 82445, Taiwan; 6 Department of Chinese Medicine, E-Da Hospital, Kaohsiung, 82445, Taiwan; 7 Department of Surgery, E-Da Cancer Hospital, Kaohsiung, 82445, Taiwan; 8 School of Medicine, I-Shou University, Kaohsiung, 82445, Taiwan

**Keywords:** SH3 domain GRB2-like endophilin B1, glioblastoma, H_2_O_2_, redox, mitochondria

## Abstract

**Objectives:**

Glioblastoma (GBM) is a prevalent malignant brain tumor prone to drug resistance. We previously found a strong correlation between SH3 domain GRB2-like endophilin B1 (SH3GLB1) and superoxide dismutase 2 (SOD2), which converts O_2_ to hydrogen peroxide (H_2_O_2_). Prior studies show that H_2_O_2_ redox signaling is vital for physiological processes and can drive tumor progression. Therefore, we aim to define how H_2_O_2_ signaling regulates SH3GLB1 and AKT (protein kinase B) pathways in GBM and to assess whether modulating H_2_O_2_ reverses temozolomide (TMZ) resistance.

**Methods:**

We used cultured cells and pharmacological inhibitors and activators to confirm the significance of H_2_O_2_ signaling. GBM cells were used to verify the role of H_2_O_2_ signaling in cell state transitions and animal experiments identified optimal treatment strategies.

**Results:**

We found that SOD2 acts as an upstream regulator of SH3GLB1. When SOD inhibitors and TMZ were combined, cells showed reduced SH3GLB1 and autophagy levels. SH3GLB1 was found to be regulated by H_2_O_2_ via AKT signaling using redox homeostasis-regulating experiments. Although treatment-induced changes in mitochondrial H_2_O_2_ levels mirrored those in the cytosol, parental and resistant cells exhibited divergent fates, highlighting cell-fate plasticity. TMZ combined with a redox modulator reduced resistant tumor cell growth (about 2/3 reduction of tumor size; *p* < 0.05) and suppressed SH3GLB1 and autophagy levels in animal models. The TMZ-induced increase in SH3GLB1 expression was reversed by HgCl_2_, which inhibited the aquaporin-9/AKT signaling.

**Conclusion:**

Overall, these findings underscore the importance of H_2_O_2_-SH3GLB1 signaling in GBM and may inform future therapeutic strategies for overcoming TMZ resistance.

## 1 Introduction

Glioblastoma (GBM) is the most common malignant primary brain tumor in adults and remains highly lethal, with frequent recurrence despite maximal safe resection followed by radiotherapy with concomitant and adjuvant temozolomide (TMZ) [1]. Known environmental risks are few, with high dose ionizing radiation as the principal risk, whereas allergic or atopic conditions have been associated with lower risk [1]. Biologically, isocitrate dehydrogenase (IDH) mutant astrocytomas differ from IDH wild type GBM, and O6-methylguanine-DNA methyltransferase (MGMT) promoter methylation predicts sensitivity to alkylating agents within a setting of pronounced intratumoral heterogeneity and recurrent pathway alterations that involve the RTK/PI3K/AKT, RAS/RAF/MEK/ERK, nuclear factor kappa B (NF-kappa B) and Transforming Growth Factor (TGF)-beta pathways [1]. Beyond DNA repair mediated by MGMT, TMZ also induces oxidative stress and reactive oxygen species (ROS) signaling [2], and adaptive antioxidant defenses, such as superoxide dismutases (SODs), catalase (CAT), and glutathione peroxidases (GPxs), are the primary system that regulates ROS levels. Superoxide dismutase isoenzymes convert superoxide to hydrogen peroxide, and catalase together with glutathione peroxidases removes hydrogen peroxide while shaping redox signaling [3]. Increasing the capacity of these enzymes can lessen therapy-induced oxidative stress and favor tumor cell survival, which can contribute to chemoresistance, whereas reducing their activity can restore drug sensitivity [4]. Notably, GPx4 governs lipid peroxidation and ferroptosis, linking antioxidant control to drug response [5]. Moreover, reduced mitochondrial DNA (mtDNA) copy number is observed in cancer and linked to decreased mitochondrial membrane potential (ΔΨm), resulting in mitochondrial dysfunction [6]. This stress activates mitochondria to nucleus retrograde signaling, which reprograms nuclear gene expression and shifts cellular states [6].

Our previous studies and others have indicated that TMZ treatment induces ROS production [2,4] in mitochondria. This enhances the antioxidant mechanism via SOD2 signaling and enriches tumor-initiating cells (TICs) features to evade the cytotoxicity of TMZ [4]. Thus, it is conceivable that resistant tumors benefit from the enrichment of specific cells with excellent ability or plasticity in managing ROS. SOD2 catalyzes the dismutation of superoxide (O_2_^.−^) into hydrogen peroxide (H_2_O_2_) and oxygen (O_2_) in the mitochondrial matrix, with H_2_O_2_ subsequently detoxified primarily by CAT and GPx [3]. H_2_O_2_ is an important secondary messenger involved in physiological signaling [3] and is associated with Akt phosphorylation and Bax activation [7]. The lifespan of H_2_O_2_ is longer than that of other ROS molecules, such as O_2_^.−^ and ·OH [8], therefore, we strongly suspect that H_2_O_2_ may be involved in the ROS-related signaling to promote cell resistance against TMZ.

SH3 domain GRB2-like endophilin B1 (SH3GLB1) is a member of the endophilin family and is called endophilin B1 or Bax-interacting factor 1 [9]. This protein is involved in many mitochondrial functions, including autophagy and membrane remodeling [10]. SH3GLB1 is also involved in directing apoptosis by modulating the permeabilization of the outer mitochondrial membrane and activating Bax to transfer apoptotic proteins from the mitochondrial intermembrane space into the cytosol [11]. SH3GLB1, a protein associated with mitochondrial dynamics and autophagy, was upregulated in treatment-resistant GBM cells. This protein mediates oxidative phosphorylation and contributes to the acquired resistance to TMZ by regulating autophagy [12].

Notably, we found a strong association between SOD2 and SH3GLB1 in clinical samples [12], suggesting that SOD2-mediated oxidative homeostasis may directly influence SH3GLB1-regulated autophagy and thereby contribute to therapy resistance. However, it remains unclear how resistance-related ROS regulation is associated with the mitochondria-related molecules involved in TMZ cytotoxicity.

As SH3GLB1 is known to regulate autophagy and mitochondrial functions, we examined whether these processes are influenced by ROS signaling (H_2_O_2_) during acquired TMZ resistance. Thus, we hypothesized that TMZ may promote the production of H_2_O_2_ in the mitochondria and transfer the signaling of H_2_O_2_ to the cytosol, where it is continuously transmitted to downstream factors and affects gene expression and cell fate.

## 2 Materials and Methods

### 2.1 Chemical Reagents

TMZ (Sigma-Aldrich, St. Louis, MO, USA; Cat No. T2577), catalase inhibitor 3-amino-1,2,4-triazole (ATZ; Sigma-Aldrich; Cat No. A8056), GPx inhibitor 4-hydroxynonenal (HNE; Cayman Chemical, Ann Arbor, MI, USA; Item No. 32100), a precursor of cysteine and glutathione that promotes GPx function, N-acetyl-L-cysteine (NAC; Sigma-Aldrich; Cat No. A7250), and HgCl2 (Sigma-Aldrich; Cat No. M1136) were used as co-treatments to modulate the ROS reaction. Carbonyl cyanide m-chlorophenyl hydrazone (CCCP; Cayman Chemical; Item No. 25458), an uncoupling agent for ΔΨm, was used in mitochondria studies. We concurrently measured the activities of catalase and GPx using assay kits (Cayman Chemical; Item No. 707002 and 703102) according to the manufacturer’s instructions. MK-2260 (Cayman Chemical; Item No. 11593), a p-AKT inhibitor, and SC-79 (2-Amino-6-chloro-α-cyano-3-(ethoxycarbonyl)-4H-1-benzopyran-4-acetic acid ethyl ester; Cayman Chemical; Item No. 14972), a p-AKT activator, were used in cell line studies. The following antibodies were used for western blot analyses: SH3GLB1 (Cat No. 15422-1-AP; 1:5000), aquaporin 9 (AQP9; Cat No. 20380-1-AP; 1:5000) (Proteintech, Rosemont, IL, USA), Microtubule-associated protein 1 light chain 3B (LC3B; Santa Cruz Biotechnology, Dallas, TX, USA; sc-376404; 1:20000), p62 (Cat No. 5114; 1:6000), SOD2 (Cat No. 13141; 1:6000), AKT (Cat No. 9272; 1:6000), p-AKT (Cat No. 9271; 1:6000) (Cell Signaling, Danvers, MA, USA), vinculin (Thermo Fisher Scientific, Waltham, MA, USA; Cat No. 14-9777-82; 1:10000), and actin (Merck Millipore, Burlington, MA, USA; Cat No. MAB1501; 1:20000).

### 2.2 Cell Culture

U87MG was purchased from the Bioresource Collection and Research Center (Hsinchu, Taiwan; BCRC number 60360) and A172 was obtained from the American Type Culture Collection (Manassas, VA, USA; Cat No. CRL-1620). Both institutions routinely perform mycoplasma testing. Cells were cultured in Dulbecco’s modified Eagle’s medium (DMEM) (Thermo Fisher Scientific; Cat No. 11995073) supplemented with 10% fetal bovine serum (FBS) and 1%–2% penicillin/streptomycin (Thermo Fisher Scientific; Cat No. 15070063). Resistant cell lines (U87MG-R and A172-R) were derived from parental U87MG and A172 cells from a previous study, and these cells were maintained following the same procedures as those used for the parental cells [4,13,14]. Primary GBM and P1S tumors, or naïve tumors, were used [4,12]. These were obtained from patients who had undergone multiple treatments or no treatment, respectively (written informed consent was obtained from the patient, and all procedures conformed to the Declaration of Helsinki), and were maintained in male NOD-SCID mice (6–7 weeks old; BioLASCO, Taipei, Taiwan; NHRI-IACUC-108048-A). All animal experimental protocols were approved by the Institutional Animal Care and Use Committee of the National Health Research Institutes The IRB (EC1021209, National Health Research Institutes and 201402018, Taipei Medical University) approved the application of the tissue array blocks and primary cells.

### 2.3 Bioinformatics Analysis

We used the Search Tool for the Retrieval of Interacting Genes/Proteins (STRING; https://string-db.org/; version 12) bioinformatics tool [15] to analyze the networks/interactions between ROS molecules and autophagy using an algorithmic assembly from the STRING database. Those genes include SOD1, SOD2, CAT, GPx1, SH3GLB1, UV radiation resistance-associated (UVRAG), Microtubule associated protein 1 light chain 3 beta (MAP1LC3B), Phosphatidylinositol 3-kinase catalytic subunit type 3 (PIK3C3), Beclin 1 (BECN1), Autophagy related 12 (ATG12), Autophagy related 5 (ATG5), Unc-51 like autophagy activating kinase 1 (ULK1). Public GBM data were accessed via GlioVis (http://gliovis.bioinfo.cnio.es/) for gene analysis. For mRNA expression, we used TCGA dataset (GBM: *n* = 528; non-tumor: *n* = 10). Expression values were retrieved as log_2_-normalized RSEM (RNA sequencing by Expectation–Maximization) [the formula is log_2_(RSEM + 1)] from GlioVis, and no additional transformation or normalization was applied.

### 2.4 Detection of Autophagosomes

The resistant cells (U87MG-R and A172-R) were seeded at 5 × 10^5^ cells per well in 6-well plates to reach 90% confluence at transfection. Cells were transfected with an LC3B-EGFP plasmid (from Dr. Li-Jin Hsu, National Cheng Kung University) or control vector using Lipofectamine (LTX with Plus, Thermo Fisher Scientific; Cat No. A12621). For each 6-well plate well, 2 μg plasmid DNA was mixed with 2 μL PLUS reagent in 150 μL Opti-MEM and combined with 6 μL Lipofectamine LTX pre-diluted in 150 μL Opti-MEM. Complexes were incubated for 10 min at room temperature and added to cells. Medium was replaced after 4 h and cells were incubated 24 h post-transfection before stimulation.

After TMZ (100 μM) stimulation or vehicle control treatment for 24 h, LC3B-EGFP puncta were imaged on a Nikon Eclipse Ti2 inverted fluorescence microscope (Nikon Corporation, Tokyo, Japan). GFP excitation was at ~488 nm and emission collected at 510–550 nm using a standard FITC/GFP filter set. LC3B-EGFP puncta were counted manually by two independent observers blinded to group allocation on the raw images. The results were reported as puncta per cell.

### 2.5 Immunohistochemistry

Tissue sections from the xenografted mice were fixed in paraformaldehyde and embedded in paraffin. Staining was performed as previously described [4]. Staining was identified after the usage of SH3GLB1, p-AKT, LC3B and p62 primary antibodies at room temperature for 1 h (DAKO HRP secondary antibodies (Agilent Technologies, Santa Clara, CA, USA) were used; anti-mouse Cat No. K4001, anti-rabbit Cat No. K4002; 1:500 dilution; at room temperature for 1 h) and evaluated through automated analysis using ImageJ software (https://imagej.net/ij/; version 1.53, National Institutes of Health, Bethesda, MD, USA). Quantification used integrated density with identical thresholds and exposure settings across samples. To minimize variability in immunohistochemistry (IHC) results, we applied standardized staining conditions and identical acquisition settings.

### 2.6 Gene Modulation

In siRNA transfection, the resistant cells (U87MG-R and A172-R) were seeded at 5 × 10^5^ cells/well in 6-well plates (90% confluence). Loss-of-function was performed with a non-targeting control siRNA and SOD2 siRNA (Thermo Fisher Scientific/Ambion; ID s13268; Cat No. 4390824) [4] or SH3GLB1 siRNA (GenePharma, Shanghai, China; sense 5^′^-GGGAAUCAGCAGUACACAUTT-3^′^, antisense 5^′^-AUGUGUACUGCUGAUUCCCTT-3^′^) [12]. For each well, siRNA-lipid complexes were prepared in Opti-MEM as follows: 3 μL of 10 μM siRNA (30 pmol) in 150 μL Opti-MEM was combined with 9 μL Lipofectamine RNAiMAX (Thermo Fisher Scientific; Cat No. 13778150) pre-diluted in 150 μL Opti-MEM, incubated 10 min at room temperature, and added dropwise to cells. Cells were incubated 72 h before protein assays and knockdown efficiency was assessed by Western blotting.

For stable modulation, the resistant cells (U87MG-R) were infected with lentiviral shRNA targeting SH3GLB1 (Academia Sinica, Taipei, Taiwan; Clone ID TRCN0000240811; sense 5^′^-CCGGTTATGGTAATGCCCTTATTAACTCGAGTTAATAAGGGCATTACCATAATTTTTG-3^′^, antisense 5^′^-AATTCAAAAATTATGGTAATGCCCTTATTAACTCGAGTTAATAAGGGCATTACCATAA-3^′^) or with an empty-vector control at multiplicity of infection (MOI) 2~5 in the presence of polybrene (Sigma-Aldrich; Cat No. TR-1003) 8 μg/mL. After 24 h, medium was replaced, and cells were selected with puromycin (Sigma-Aldrich; Cat No. P8833) 1 μg/mL for 72 h. The efficiency of knockdown was verified by Western blotting for SH3GLB1 relative to control.

Gain-of-function in the parental cells (U87MG and A172) was achieved by transfecting a SH3GLB1 overexpression plasmid (GenScript Biotech, NJ, USA; Clone_ID 20190603) or matched empty vector using Lipofectamine LTX with PLUS reagent (Thermo Fisher Scientific; Cat. A12621). For each 6-well, 1 μg DNA was mixed with 1.0 μL PLUS in Opti-MEM (150 μL) and combined with 3.0 μL Lipofectamine LTX in Opti-MEM (150 μL). The complexes were incubated 10 min at room temperature and added to cells. The medium was replaced after 4 h, and cells were analyzed 72 h post-transfection. Overexpression efficiency was evaluated by Western blotting.

### 2.7 Western Blot Analysis

Cells were washed twice with ice-cold PBS and lysed on ice in RIPA lysis buffer (Merck Millipore; Cat No. 20-188) overnight. The stock formulation is 0.5 M Tris-HCl (pH 7.4), 1.5 M NaCl, 2.5% sodium deoxycholate, 10% NP-40, 10 mM EDTA and was diluted to 1X working strength prior to use. Lysates were clarified by centrifugation (12000× *g*; 10 min; 4°C), and supernatants were collected. Protein concentration was determined by a colorimetric assay using a bovine serum albumin (BSA) standard curve; absorbance was read at 550 nm on a PowerWave 340 microplate spectrophotometer (BioTek, Winooski, VT, USA). Sample concentrations were calculated from the standard curve, and 30 μg total protein per lane was used for SDS-PAGE.

Cell lysates were separated using SDS-PAGE (8%~12% gel; loading 30 μg protein per lane) and transferred to polyvinylidene difluoride membranes (Merck Millipore; Cat No. IPVH85R). Next, the membranes were blocked with 5% nonfat milk (room temperature for 1 h) and incubated overnight (4°C) with primary antibodies, detecting levels of SH3GLB1, SOD2, p-AKT, AKT, LC3B, p62, AQP9, vinculin and actin. The cells were then washed and incubated with secondary antibodies (Goat anti-Rabbit antibody, Merck Millipore; Cat No. AP132P, 1:5000–1:10000 dilution; Goat anti-mouse antibody, BioLegend, San Diego, CA, USA, Cat No. 405306, 1:3000–1:6000 dilution; the incubation time is 1 h at room temperature). After eliciting signals with a chemiluminescent substrate, Amersham Hyperfilm ECL (GE Healthcare, Chicago, IL, USA; Cat No. 45-000-999) was used to detect expression intensity.

### 2.8 Detection of H_**2**_O_**2**_ Production

H_2_O_2_ concentration was determined using the Amplex Red Hydrogen Peroxidase Assay (Thermo Fisher Scientific; Cat No. A22188). A detection probe, H_2_DCFDA (2^′^,7^′^-dichlorodihydrofluorescein diacetate, Thermo Fisher Scientific; Cat No. D399), was used for the ROS staining. Preparation was performed according to the manufacturer’s instructions. Cells were incubated with H_2_DCFDA at 10 μM for 30 min at 37°C in the dark, then washed and analyzed as described.

### 2.9 Cell Density Assay

Cells were cultured at a density of 20,000 cells/mL/well in a 24-well plate, with or without treatment. Cells were treated with the indicated concentrations of TMZ (100 μM) and other drugs (ATZ (20 mM); HNE (10 μM); NAC (10 mM)). The cells were then stained with 0.5% methylene blue (Sigma-Aldrich; Cat No. M9140) and 1% N-lauroyl-sarcosine (Sigma-Aldrich; Cat No. 61745), and absorbance was read at 570 nm on a PowerWave 340 microplate spectrophotometer (BioTek). Background was subtracted using dye-only blanks: A570 (corrected) = A570 (sample) − A570 (blank); Cell viability (%) = 100 × [A570 (treated group) − A570 (blank)]/[A570 (control group) − A570 (blank)].

### 2.10 Tumor Xenograft Model

For cell-derived xenografts, all experimental animal protocols were approved by the Institutional Animal Care and Use Committee of the National Health Research Institute (NHRI-IACUC-108048-A), and all animal experiments were designed and reported in accordance with Animal Research: Reporting of In Vivo Experiments (ARRIVE) guidelines (https://arriveguidelines.org). The use of clinical tumor samples was approved by the IRB of EC1021209, National Health Research Institutes and 201402018, Taipei Medical University. A fresh tumor sample was obtained from a patient with GBM after surgery. Written informed consent was obtained from the patient, and all procedures conformed to the Declaration of Helsinki. The tumors were minced and subcutaneously implanted into the right flank of NOD-SCID mice. When the tumors reached an appropriate size, the mice were sacrificed for tumor extraction. The tumor pieces, such as P1S tumor (from a recurrent patient mentioned above), were minced and enzymatically dissociated with the Papain Dissociation Kit (Miltenyi Biotec, Bergisch Gladbach, Germany; Cat No. 130-095-942) on a gentleMACS™ dissociator. Mouse cells were removed by negative magnetic selection using the Mouse Ablation Kit (Miltenyi Biotec; Cat No. 130-104-694). The purified tumor cells (2 × 10^6^ in 20 μL) were injected subcutaneously into the right flank of NOD-SCID mice. When xenografts reached ~200 mm^3^, mice (N = 3~5 in each group) were treated with vehicle or TMZ (5 mg/kg per day) for 3 weeks, and tumors were then collected for Western blotting analysis of protein expression.

Moreover, male NOD-SCID mice were subcutaneously injected with U87MG-R-luciferase-EGFP cells [5] with or without SH3GLB1 knockdown. Cells at a density of 2 × 10^6^/20 μL were inoculated into the flanks. On day ten, the animals (N = 5 in each group) were randomly assigned to the treatment groups (TMZ, 5 mg/kg; HNE: 2.5 mg/kg) after injecting the cells. The frequency of dosing is once a day. Tumor growth was measured and recorded on the indicated days by *in vivo* bioluminescence imaging using an IVIS 200 imaging system (Xenogen/Caliper Life Sciences, Alameda, CA, USA; Living Image software, version 3.2) after the mice were intraperitoneally injected with firefly luciferase substrate (80 mg/kg in saline; Promega, Madison, WI, USA; Cat No. P1041). A fixed region of interest (ROI) was drawn around each tumor, and a background ROI was placed over non-tumor tissue. To compare longitudinal growth within animals, total photon flux (photons per second) was exported from Living Image and corrected by subtracting background following the formula: Flux_corrected = Flux_ROI − Flux_background. Finally, the tumors were extracted after sacrifice to measure tumor weight, and IHC staining and western blotting analysis were performed.

Animals were monitored daily for body weight and general health. Humane endpoints were predefined as greater than 20% loss of body weight, impaired mobility, or signs of distress, and animals that met these criteria were euthanized by carbon dioxide inhalation in accordance with institutional IACUC policy.

### 2.11 Statistics

Statistical analyses were performed using the Prism 7 software (GraphPad, La Jolla, CA, USA). Continuous variables were assessed for differences by using an unpaired two-tailed Student’s *t*-test. Statistical significance was set at *p < 0.05, **p < 0.01 and ***p < 0.001.

## 3 Results

### 3.1 SOD2 Affects the Expression of SH3GLB1

**Figure 1:**
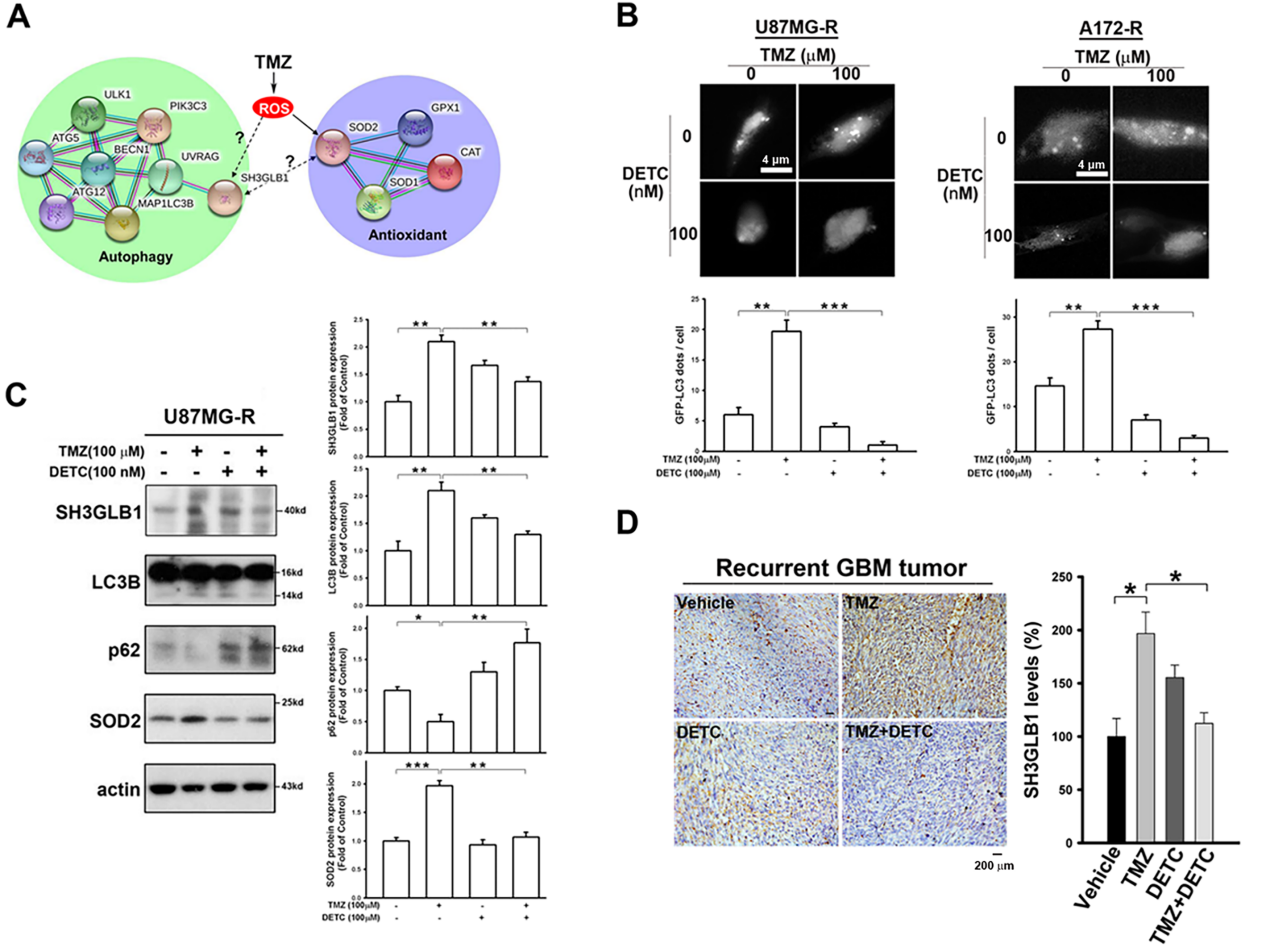

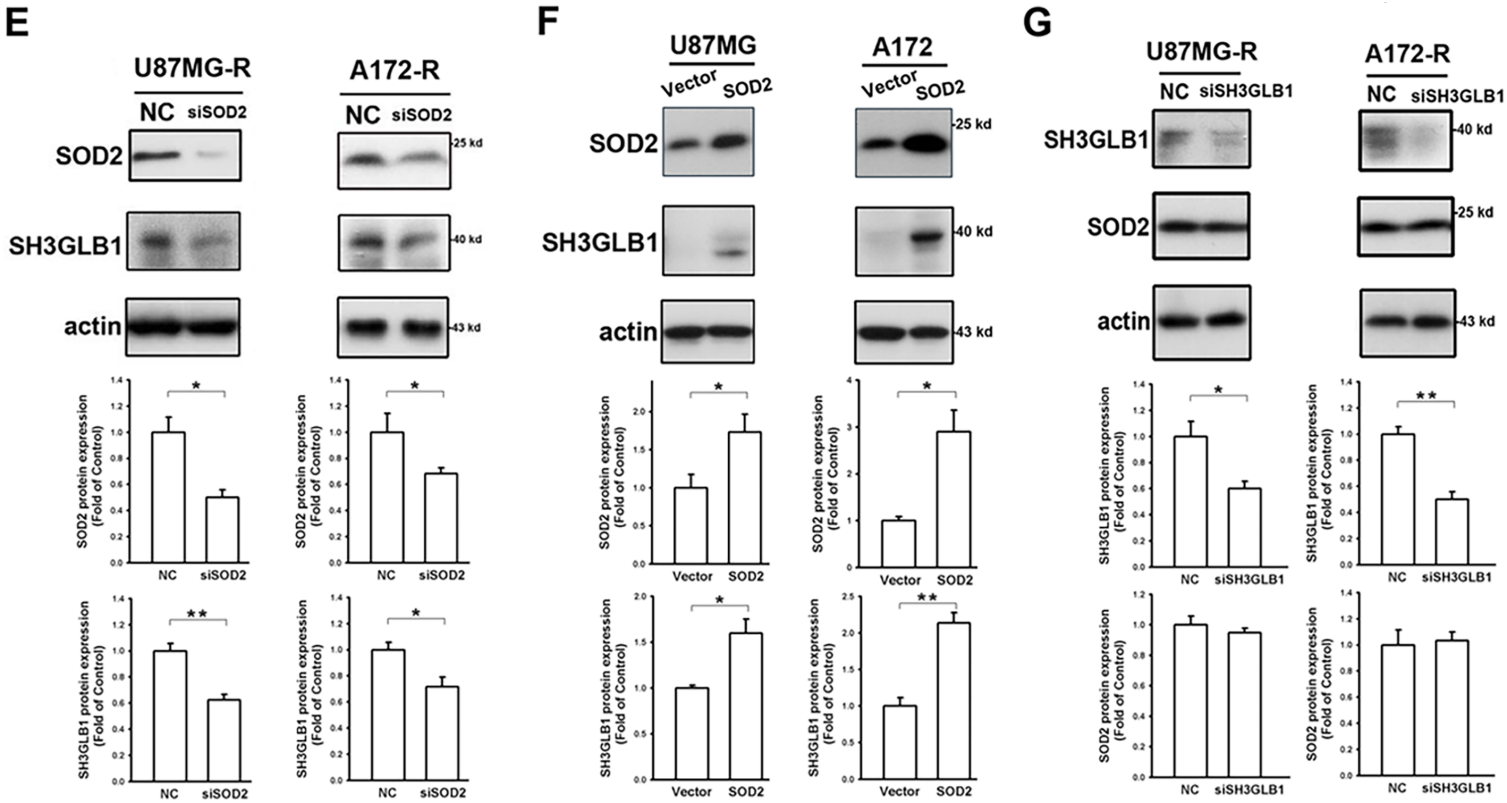
SH3 domain GRB2-like endophilin B1 (SH3GLB1) is a downstream protein of superoxide dismutase 2 (SOD2). (**A**) The biological network of SH3GLB1-mediated autophagy and SOD2-involved antioxidants was predicted using the STRING bioinformatics tool. (**B**) Resistant cells were transfected with the microtubule-associated protein 1 light chain 3-Enhanced Green Fluorescent Protein (LC3-EGFP) plasmid, and representative fluorescent images for the formation of LC3-EGFP dots (puncta) are shown. Scale bar: 4 μm. (**C**) Protein immunoblotting showing that the resistant cells treated with temozolomide (TMZ) for 24 h induced autophagic reactions, which were attenuated by pretreatment with diethyldithiocarbamic acid (DETC; an SOD inhibitor). (**D**) Immunohistochemical (IHC) staining demonstrating SH3GLB1 expression in primary, recurrent glioblastoma (GBM-R) cells inoculated subcutaneously into mice receiving the indicated treatments for 15 days. A statistical graph is shown in the right-hand panel. Scale bar: 200 μm. (**E**) SOD2 siRNA or (**F**) overexpression vectors were used in U87MG- and A172-resistance cells or U87MG- and A172-parental cells, respectively, three days after transfection to examine the association between SOD2 and SH3GLB1. (**G**) SH3GLB1 siRNA was used in U87MG- and A172-resistance cells, and the association between SH3GLB1 and SOD2 was studied using western blotting. For each blot, the adjacent bar chart depicts the fold change relative to control. *N* = 3 in each group. *p < 0.05, **p < 0.01 and ***p < 0.001

**Figure A1:**
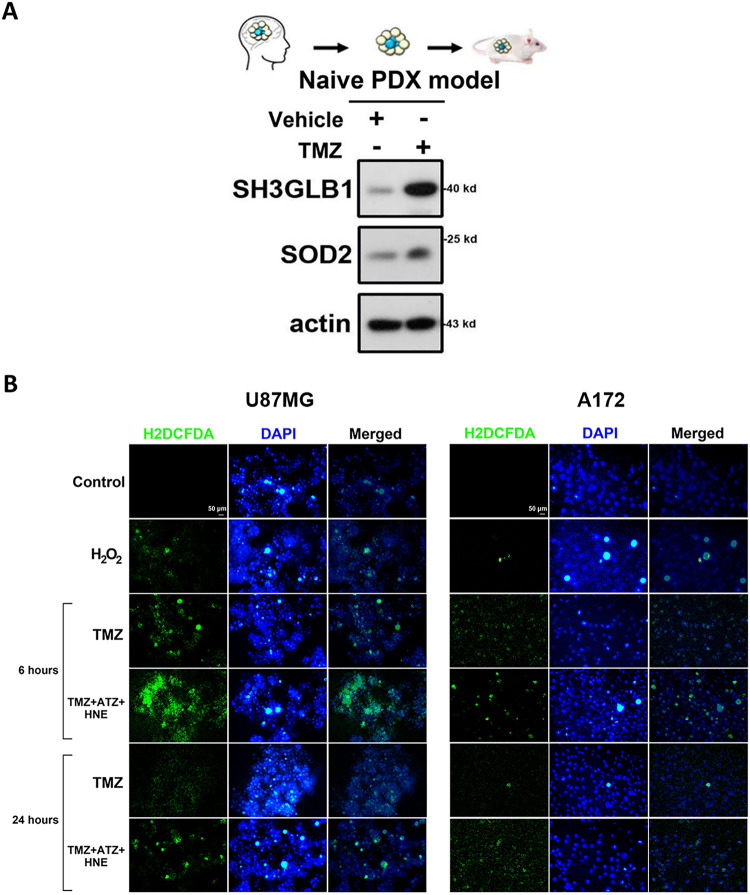
(**A**) SH3 domain GRB2-like endophilin B1 (SH3GLB1) and superoxide dismutase 2 (SOD2) levels were simultaneously enhanced in the patient derived xenografts (PDX) model of naïve glioblastoma (GBM) tumors after temozolomide (TMZ) (5 mg/kg) treatment for three weeks. (**B**) The results show increased levels of 2^′^,7^′^-dichlorodihydrofluorescein diacetate (H_2_DCFDA) staining (a reactive oxygen species (ROS) detection probe) in parental U87MG (for 6 h) or A172 (for 24 h) cells after triple co-incubation with TMZ, ATZ, and HNE. H_2_O_2_: 100 μM. Scale bar: 50 μm

Previously, we found that increased SH3GLB1 levels in GBM cells were associated with TMZ resistance and SOD2 expression [12]. TMZ induced a simultaneous increase in SH3GLB1 and SOD2 protein levels in naïve primary tumor cells (Fig. A1A). We performed bioinformatics analysis of protein interactions using the STRING tool and database to further understand this association. As shown in Fig. 1A, the relationships between SH3GLB1-mediated autophagy, ROS production, and SOD2 remain unclear. Therefore, we investigated whether SH3GLB1 was associated with ROS or SOD2. SOD2 regulates ROS production during the acquisition of TMZ resistance in GBM cells [4]. We hypothesized that altered SOD2 was responsible for SH3GLB1 expression in resistant cells to explore the role of the SOD2-axis in acquired GBM resistance to TMZ.

Diethyldithiocarbamic acid (DETC) was used to inhibit protein function to study the impact of SOD2. TMZ-induced Microtubule-associated protein 1 light chain 3-Enhanced Green Fluorescent Protein (LC3-EGFP) puncta formation was alleviated by co-treatment with DETC (Fig. 1B). Immunoblot analysis showed that combination treatment reduced SH3GLB1 levels and inhibited autophagy signaling (Fig. 1C). Decreased SH3GLB1 expression was also observed in *in vivo* tumors that were subcutaneously implanted with primary resistant GBM cells (P1S) and co-treated with TMZ and DETC (Fig. 1D). These results indicated that SOD2 inhibition could regulate TMZ-induced autophagy signaling. SOD2 knockdown and overexpression resulted in reduced and increased levels of SH3GLB1 in resistant and parental cells, respectively (Fig. 1E,F). In contrast, SH3GLB1 knockdown did not alter SOD2 expression (Fig. 1G). These results indicated a regulatory role for SOD2 in SH3GLB1 expression.

### 3.2 Hydrogen Peroxide Regulates SH3GLB1 Expression in Parental GBM Cells

**Figure 2:**
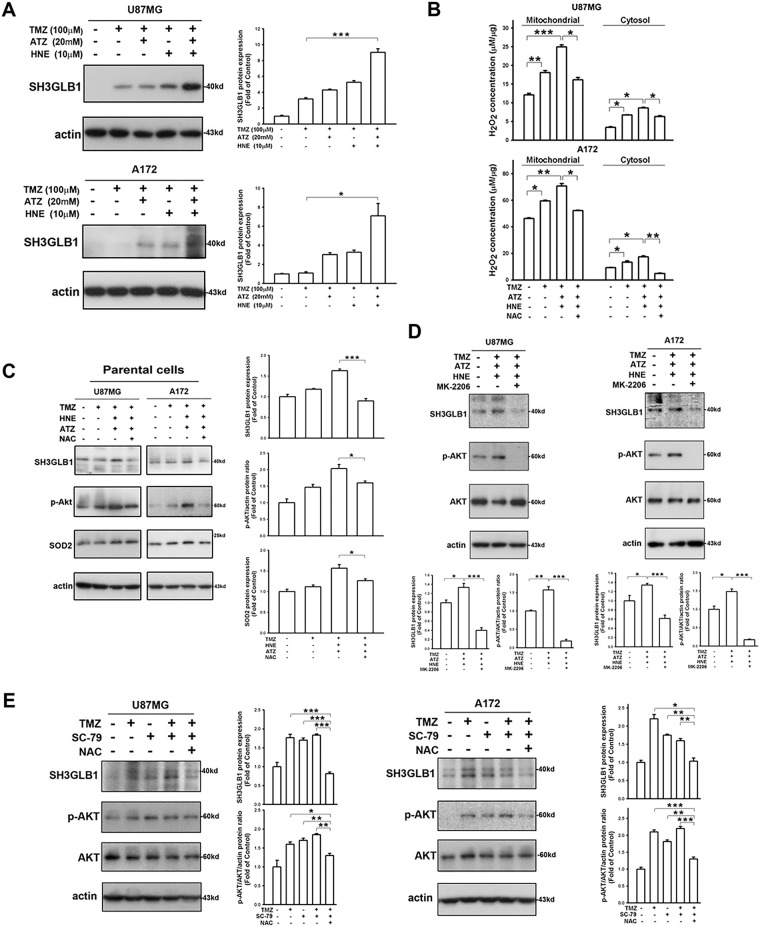
SH3GLB1 levels are regulated by hydrogen peroxide. (**A**) Blots show that co-treatment with TMZ + 4-hydroxynonenal (HNE) + 3-amino-1,2,4-triazole (ATZ), enhanced the expression of SH3GLB1 in parental cells. (**B**) Cells were pretreated with N-acetyl-L-cysteine (NAC) and subjected to three co-treatments. U87MG cells were co-treated for 8 h and A172 cells for 18 h. Intracellular H_2_O_2_ levels were measured, and (**C**) SH3GLB1, p-AKT (Ser473), and SOD2 levels were detected by western blotting. (**D**) MK-2206 inhibits p-Akt (Ser473) expression. (**E**) Blots showing the levels of the indicated proteins after co-treatment with TMZ, SC-79 (2-Amino-6-chloro-α-cyano-3-(ethoxycarbonyl)-4H-1-benzopyran-4-acetic acid ethyl ester), and NAC. TMZ: 100 μM, ATZ: 20 mM, HNE: 10 μM, NAC: 10 mM, MK-2206: 5 μ, SC-79: 10 μg/mL. For each blot, the adjacent bar chart depicts the fold change relative to control. *N* = 3 in each group. *p < 0.05, **p < 0.01 and ***p < 0.001

Next, we investigated the effect of H_2_O_2_, a product of SOD2 catalysis, on the expression of SH3GLB1. As shown in Fig. 2A, SH3GLB1 expression was enhanced in the triple treatment groups (TMZ + ATZ + HNE). Upon co-incubation with ATZ and HNE, the catalytic reaction of SOD2 was blocked, resulting in increased levels of H_2_O_2_ in the mitochondria and cytosol (Fig. 2B). Enhanced ROS levels were detected after treatment (Fig. A1B). The addition of N-acetylcysteine (NAC), an antioxidant that enhances GPx function, attenuated the expression of H_2_O_2_ (Fig. 2B). Western blotting revealed that increased H_2_O_2_ levels were associated with enhanced SH3GLB1 and p-AKT levels in parental U87MG and A172 cells (Fig. 2C). The upregulation of SH3GLB1 by the combination of TMZ, ATZ, and HNE was inhibited by the p-AKT inhibitor MK-2206 (Fig. 2D). Moreover, the increase in SH3GLB1 expression following treatment with TMZ and the p-AKT activator SC-79 was reversed by NAC treatment (Fig. 2E). These results indicated that H_2_O_2_ regulated SH3GLB1 via p-AKT signaling.

### 3.3 Opposite H_**2**_O_**2**_ Responses in the Resistant Cells

**Figure 3:**
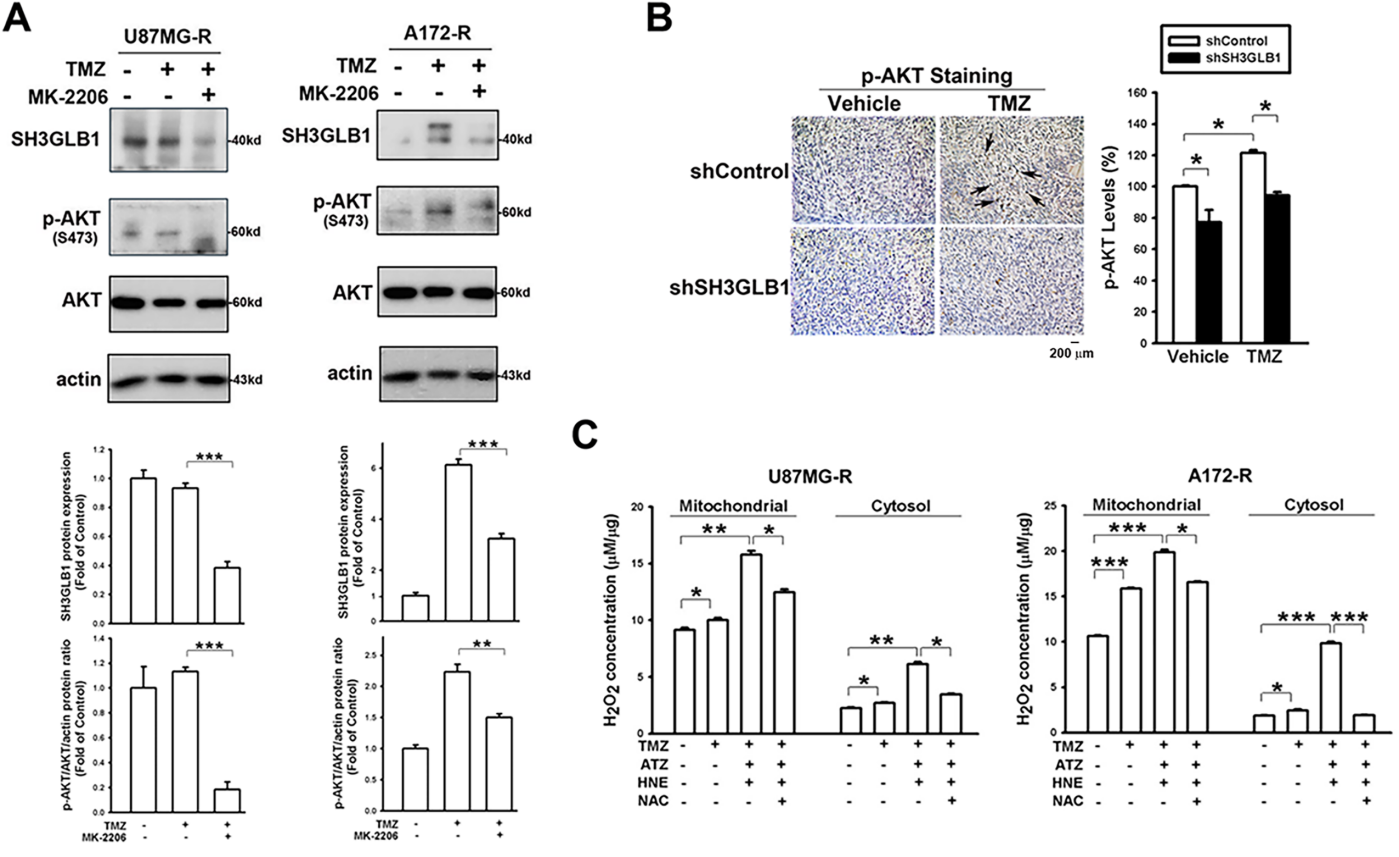

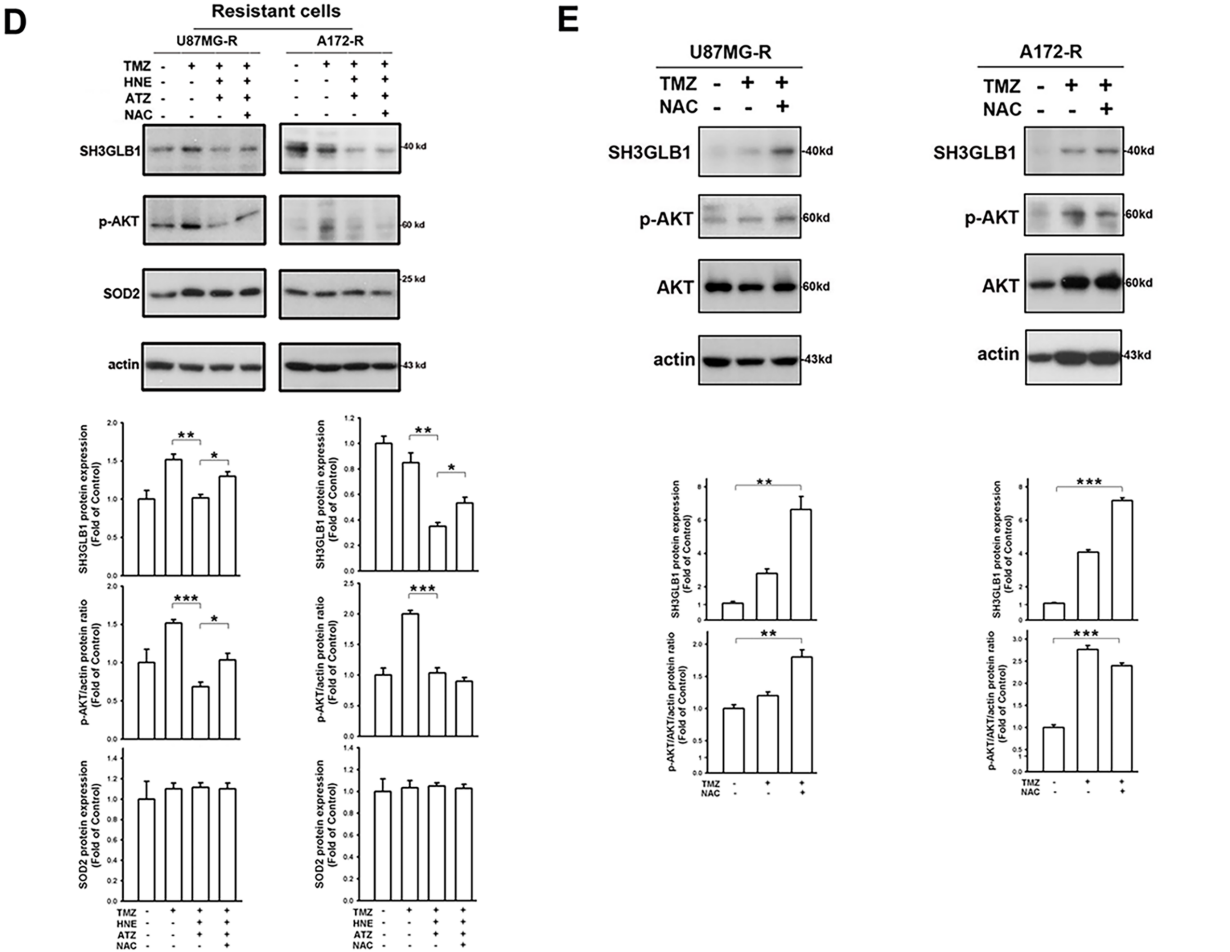
Increased levels of hydrogen peroxide demonstrate different effects on SH3GLB1 expression in the resistant cells. (**A**) MK-2206 was pretreated in the TMZ-treated resistant cells. (**B**) IHC staining demonstrating p-AKT levels in U87MG-R cells transfected with shSH3GLB1 or shControl vectors and inoculated subcutaneously into mice receiving the indicated treatments for 23 days. A statistical graph is shown in the right-hand panel. The arrows indicate the positive staining of SH3GLB1. Scale bar: 200 μm. (**C**) The resistant cells were treated with the indicated reagents. U87MG-R cells were co-treated for 18 h and A172-R cells for 8 h. The levels of intracellular H_2_O_2_ were measured. (**D**) Protein immunoblotting after stimulation and rescue treatments. (**E**) Resistant cells were co-treated with TMZ and NAC. TMZ: 100 μM, MK-2206: 5 μM, ATZ: 20 mM, HNE: 10 μM, NAC: 10 mM, MK-2206: 5 μM. For each blot, the adjacent bar chart depicts the fold change relative to control. *N* = 3 in each group. *p < 0.05, **p < 0.01 and ***p < 0.001

**Figure A2:**
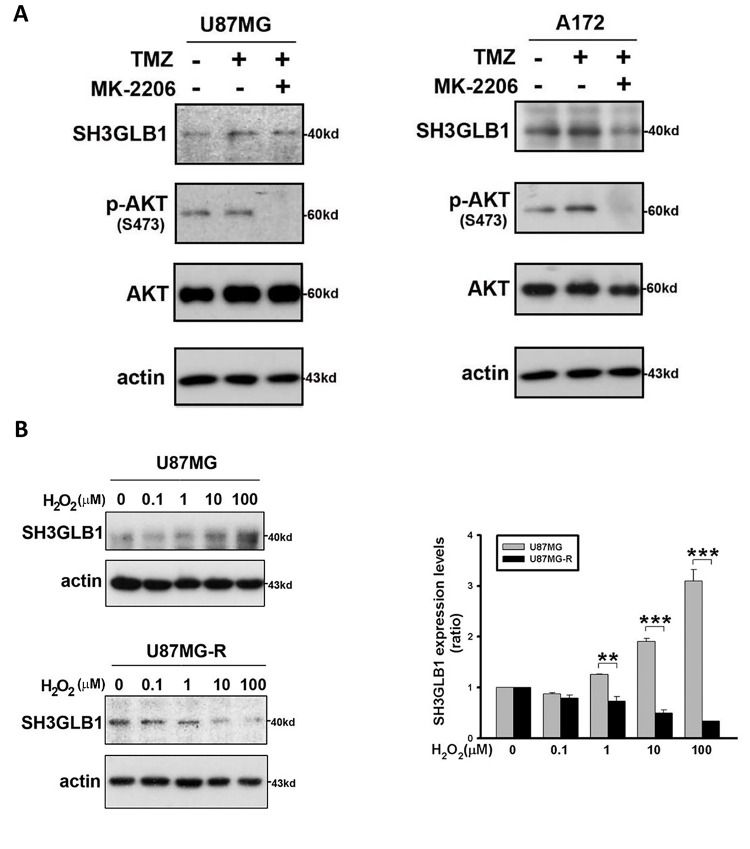
(**A**) Blots showing the levels of the indicated proteins. Resistant cells were treated with TMZ with or without MK-2206. TMZ: 100 μM, MK-2206: 5 μM (**B**) The parental and resistant cells are treated with increasing concentrations of extracellular H_2_O_2_ for 24 h. H_2_O_2_ concentrations are indicated. The summary graph demonstrates the difference in the expression of SH3GLB1 in response to extracellular H_2_O_2_ between the two cell lines. **p < 0.01 and ***p < 0.001

We further examined whether there was a similar regulatory mechanism in resistant cells. TMZ-induced SH3GLB1 expression in resistant cells was suppressed by co-treatment with a p-AKT inhibitor (Fig. 3A), and identical results were observed in parental cells (Fig. A2A). The *in vivo* studies also showed that activated p-AKT signaling was reversed following SH3GLB1 downregulation in resistant cells (Fig. 3B). SH3GLB1 and p-AKT levels were suppressed when intracellular H_2_O_2_ was increased (Fig. 3C,D), and these effects were reversed by NAC treatment. Notably, co-treatment with TMZ and NAC enhanced SH3GLB1 and p-AKT levels (Fig. 3E). Indeed, opposite responses to changes in H_2_O_2_ levels were observed in parental and resistant cells. Extracellular H_2_O_2_ was used to examine these findings. The results also showed that extracellular H_2_O_2_ dose-dependently increased the levels of SH3GLB1 in parental cells and suppressed their expression in resistant cells (Fig. A2B).

### 3.4 TMZ Combined with the ROS Modulator Affects the Resistant Cell Fate

**Figure 4:**
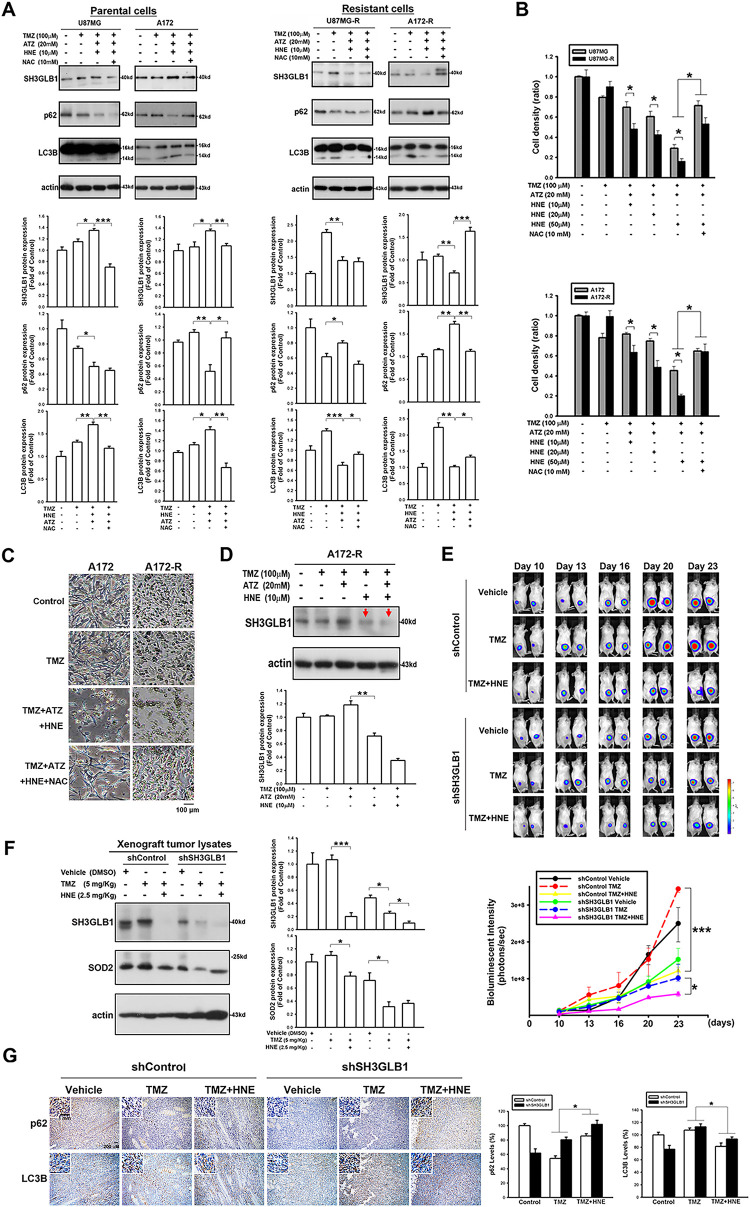
TMZ combined with the inhibitors of an H_2_O_2_-related enzyme affects autophagy levels and tumor growth in the resistant cells. (**A**) The triple co-treatment caused simultaneous changes in autophagy and SH3GLB1 expression. (**B**) Proliferation assay results for parental or resistant cells treated with the indicated compounds are shown as bar graphs, suggesting that the resistant cells were more susceptible to H_2_O_2_ accumulation. (**C**) Morphology of A172 and A172-R cells after 72 h of treatment. Control group is no TMZ-treated group. Scale bar: 100 μm. (**D**) Arrows indicate the inhibition of SH3GLB1 levels in the TMZ+HNE and TMZ+HNE+ATZ groups. (**E**) The mice were subcutaneously injected with luciferase-expressing U87MG-R cells. Bioluminescence signals were recorded on the indicated days using an IVIS imaging system. The growth curves of the tumors were analyzed according to the bioluminescence intensity. (N = 5 in each group) (**F**) Immunoblots showing the protein levels of xenograft tumor lysates from mice harvested after the indicated treatments. TMZ: 5 mg/kg, HNE: 2.5 mg/kg (**G**) The IHC staining demonstrates autophagy levels in shSH3GLB1 or shControl group of U87MG-R cells subcutaneously injected in mice and those receiving the indicated treatments. The statistic graph is shown in the right panel. Scale bar: 200 μm. Scale bar in the enlarged graph represents 1 mm. For each blot, the adjacent bar chart depicts the fold change relative to control. *N* = 3~5 in each group. *p < 0.05, **p < 0.01 and ***p < 0.001

**Figure A3:**
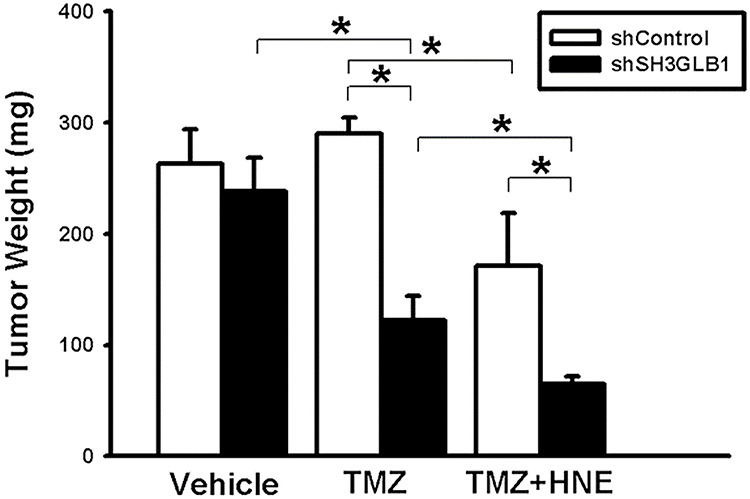
Tumor weight was measured on day 23 in the indicated groups of mice subcutaneously inoculated with U87MG-R cells (shControl or shSH3GLB1) following the administration of TMZ+HNE. *p < 0.05

Unlike in the parental cells, the diverse consequences of increased intracellular H_2_O_2_ in the resistant cells caused the cells to fail to induce autophagy (Fig. 4A). Moreover, regardless of cell density (Fig. 4B) or morphology (Fig. 4C), co-incubation of resistant cells with ATZ and HNE induced more significant TMZ cytotoxicity than the parental cells, suggesting that the resistant cells failed to induce SH3GLB1 (Fig. 4D). Mice were subcutaneously injected with U87MG-R cells carrying luciferase and infected with shRNA lentiviral vectors to investigate this. Co-treatment with TMZ and HNE showed the inhibition of luminescence intensity in the shSH3GLB1 group (Fig. 4E). The tumor weight measurements (Fig. A3) also indicate that, regardless of whether the resistant cells were transfected with shSH3GLB1, the tumors in the TMZ + HNE group were significantly smaller than those in the TMZ-only group. This finding is consistent with the luminescence data and confirms the tumor growth inhibition achieved by TMZ + HNE. Moreover, following TMZ + HNE treatment, we observed decreased levels of both SH3GLB1 (Fig. 4F) and LC3B, along with a significant increase in p62 (Fig. 4G). These findings indicate that TMZ + HNE reduces SH3GLB1 expression and autophagy *in vivo*. Collectively, the downregulation of SH3GLB1 in resistant cells retained their susceptibility to TMZ using an ROS modulator.

### 3.5 Increased Levels of Hydrogen Peroxide Derived from Damaged Mitochondrial Functions Affect SH3GLB1 Expression via AKT Signaling

**Figure 5:**
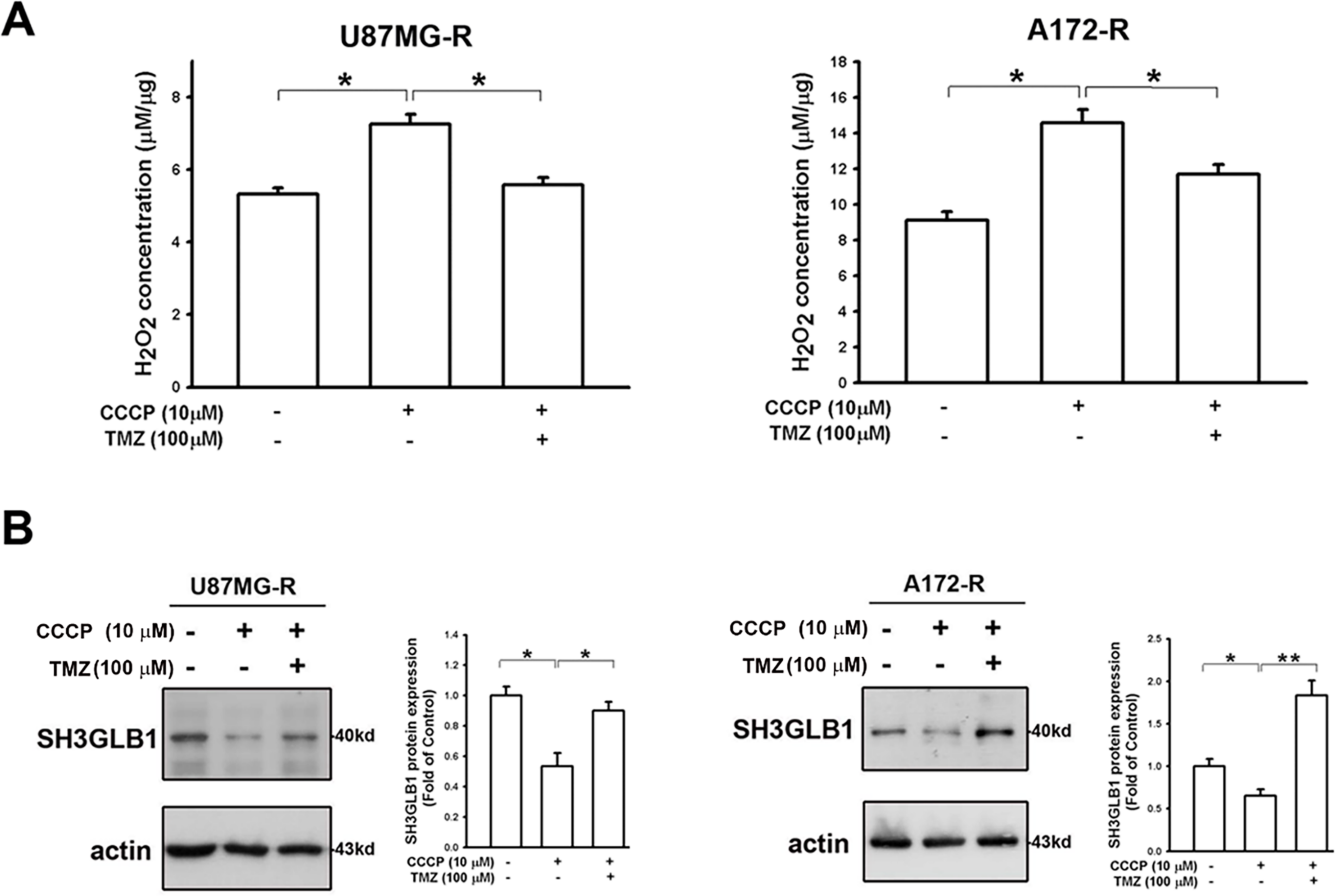

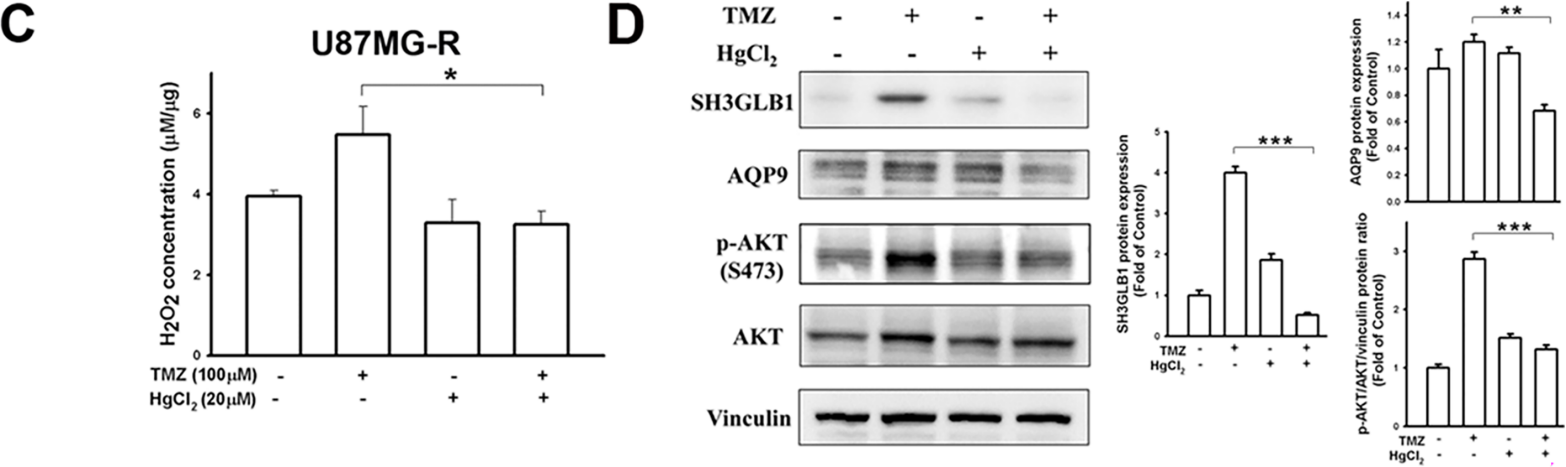
Mitochondrial dysfunction affects SH3GLB1 expression via H_2_O_2_/AKT signaling. (**A**) H_2_O_2_ levels in resistant cells were measured after the indicated treatments. TMZ: 100 μM, CCCP: 10 μM (**B**) The protein immunoblot shows that SH3GLB1 levels are regulated by treating with CCCP for 24 h with or without TMZ. Resistant cells (U87MG-R) were treated with TMZ with or without HgCl_2_. (**C**) The statistic graph shows H_2_O_2_ levels in the indicated treatments. (**D**) The blots demonstrate the indicated protein expression after the treatments. The statistic graphs are shown. TMZ: 100 μM, HgCl_2_: 20 μM. For each blot, the adjacent bar chart depicts the fold change relative to control. *N* = 3 in each group. *p < 0.05, **p < 0.01 and ***p < 0.001

**Figure A4:**
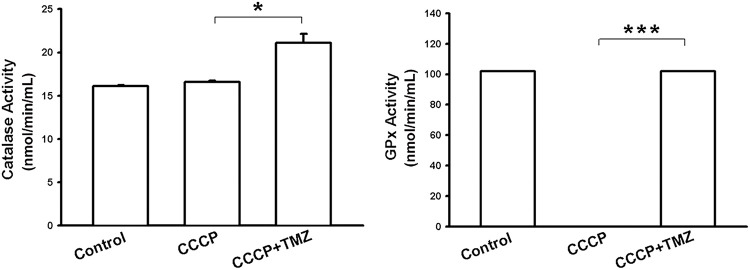
Bar graphs show catalase and GPx activity in resistant cells following the indicated treatments. *p < 0.05 and ***p < 0.001

**Figure A5:**
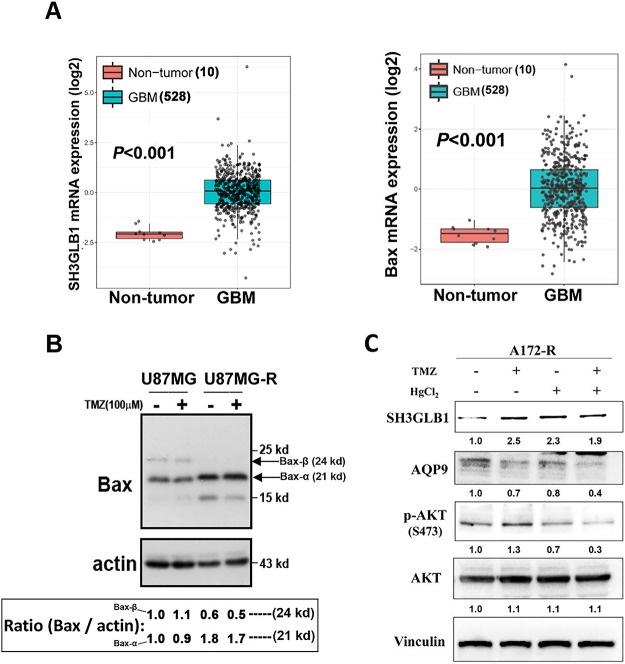
(**A**) SH3GLB1 and Bax levels were compared between normal and GBM tissues from the TCGA-GBM dataset. (**B**) The protein immunoblots showed levels of Bax-α (21 kd; the lower arrow) and Bax-β (24 kd; the upper arrow) in the parental cells and the derived resistant cells. Corresponding fold-change values (relative to control) are shown in the lower box. (**C**) The western blotting showed that the resistant cells (A172-R) were treated with TMZ with or without HgCl_2_. TMZ: 100 μM, HgCl_2_: 20 μM. Corresponding fold-change values (relative to control) are shown beneath the Western blot panels. *N* = 3 in each group

Considering that SH3GLB1 regulated the polarization of ΔΨm [16] and impairment of ΔΨm upregulated ROS in glioma cells [17], we assumed the ROS derived from ΔΨm damage could also affect SH3GLB1 expression. Our results showed that CCCP increased endogenous H_2_O_2_ production, which was reduced by co-treatment with TMZ (Fig. 5A). Notably, CCCP inhibited the GPx activity, whereas TMZ upregulated it (Fig. A4). CCCP inhibited SH3GLB1 levels in resistant cells, and these effects were reversed by co-treatment with TMZ (Fig. 5B). TMZ increased H_2_O_2_ levels, and activated phospho-AKT and enhanced SH3GLB1 levels, which were reversed by HgCl_2_ co-treatment via AQP9 regulation (Figs. 5C,D and A5C). These results demonstrated that ROS imbalance following mitochondrial damage regulates SH3GLB1 levels via endogenous H_2_O_2_ production and AKT signaling.

## 4 Discussion

SH3GLB1 promotes apoptosis by directly regulating Bax [18]. The Bax protein level increased in resistant diseases, and the tumors expressed higher levels of Bax than normal tissues in GBM TCGA data (Fig. A5A). In our model, Bax-α was upregulated while Bax-β was significantly downregulated, suggesting imbalanced activation to initiate apoptosis (Fig. A5B) [19,20]. These results suggested that the protective mechanism of SH3GLB1 overshadowed related apoptosis in resistant GBM.

Beyond the genotoxic effects, TMZ influences cellular redox homeostasis through mitochondrial ROS production [21–23]. Electron leakage from the respiratory chain generates superoxide that is dismutated by SOD2 to H_2_O_2_, enabling redox signaling but also contributing to oxidative injury when detoxification is insufficient. Reports in GBM indicated that mitochondrial ROS can be coupled to proteostasis pathways such as chaperone-mediated autophagy, with consequences for TMZ sensitivity and resistance [21]. Together, these observations support a model in which mitochondrial superoxide and derived H_2_O_2_ may be central modulators of TMZ responses in GBM. Moreover, mitochondrial programs shape drug response in GBM cells [24,25]. Increased biogenesis and oxidative phosphorylation help the cells survive treatment and blocking these pathways or disrupting dynamics controlled by DRP1 and MFN can sensitize cells to TMZ [24]. Mitophagy mediated by PINK1 and Parkin removes damaged mitochondria and is closely linked to ΔΨm [25]. Transient loss of ΔΨm can activate mitophagy and support cell survival, whereas sustained loss triggers apoptosis [26]. Therefore, tumor cells that preserve or quickly restore ΔΨm tend to be more drug resistant [26].

In our previous study, resistant GBM cells with high SOD2 levels demonstrated malignant characteristics in response to TMZ [4]. This protein has been recognized for its specific role in mitochondria, where it regulates oxidative stress [27]. ROS levels in resistant cells are lower due to the upregulation of SOD2 [4] and glutathione reductase [28]. In the present study, enhanced SH3GLB1 expression could be suppressed by co-treatment with DETC and TMZ (Fig. 1) or SOD2 RNA interference. Notably, in untreated (vehicle) cells, minimal LC3 puncta were observed, indicating a low basal level of autophagy. In contrast, TMZ treatment significantly increased puncta, reflecting its ability to induce autophagy via heightened ROS production. DETC, an SOD inhibitor that elevates superoxide, does not by itself produce sufficient oxidative stress to reduce or substantially alter baseline autophagy. However, in combination with TMZ, whose ROS-mediated stress already triggers robust autophagy, DETC shifts the redox environment such that TMZ-induced LC3 puncta formation is alleviated (Fig. 1). We thus identified downstream SH3GLB1 to be involved in the ROS-SOD2 axis (Figs. 2 and 3) in GBM cells, supporting the endogenous H_2_O_2_ reaction data (Fig. 5).

Our previous studies have shown that GBM-resistant cells have high levels of SH3GLB1, leading to TMZ resistance by enhancing autophagy-mediated oxidative phosphorylation [12]. In this study, we demonstrated that SH3GLB1-mediated autophagy was regulated by an SOD inhibitor (Fig. 1), indicating the association between ROS production and autophagy. Notably, AQP8 and AQP9 are localized to mitochondria [29], with AQP9 also implicated in astrocytoma [30]. By contrast, AQP3 resides in the plasma membrane and facilitates extracellular H_2_O_2_ uptake, raising intracellular ROS levels and activating downstream pathways such as AKT/mTOR [31]. Major stimuli like EGF and CXCL12 promote extracellular H_2_O_2_ generation via NADPH oxidase 2, thereby enhancing AQP3 function [32]. Furthermore, AQP3 expression is driven by EGF/EGFR signaling [33,34], and since TMZ can induce EGFR ligand production [35], it may also promote the transfer of extracellular H_2_O_2_ from the plasma membrane to the cytosol. Once inside the cell, this extracellular H_2_O_2_, together with endogenous H_2_O_2_, could collectively modulate AKT signaling. Further investigation is warranted to validate this mechanism.

In our previous analysis of the oxygen consumption rate [12], we noticed that spare respiration capacity (SRC), an indicator of the ability of cells to overcome severe cellular stress, was reduced in resistant cells treated with TMZ, and this was reversed as SH3GLB1 was downregulated. Furthermore, tumor cells are less capable of metabolizing H_2_O_2_ than normal cells [36], and higher H_2_O_2_ levels reduce SRC, which can be reversed by regulating ROS levels [37]. This was also evident in our model with SH3GLB1 levels, and the survival of resistant and primary cells showed different responses to H_2_O_2_-induced oxidative stress (Fig. 4). These findings suggested that resistant cells lack the cellular flexibility to adapt to rapid and intense ROS changes.

One of the roles of H_2_O_2_ is to deliver redox signals to alter cellular physiology and metabolism [38]. Increased H_2_O_2_ expression alters the ΔΨm [39] and induces TMZ resistance, causing mitochondrial remodeling [40]. CCCP, which disturbs the ΔΨm, increased H_2_O_2_ levels simultaneously with SH3GLB1 reduction, and TMZ addition could reverse this condition (Fig. 5). In terms of redox homeostasis, normal and malignant cells differentiate at thresholds to utilize or tolerate increased ROS or H_2_O_2_ levels [41,42].

**Figure 6:**
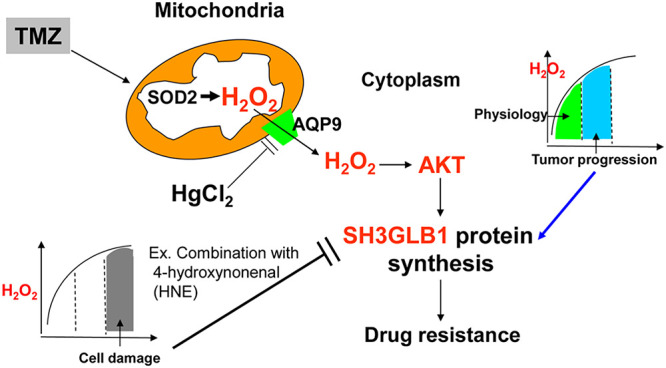
TMZ elevates reactive oxygen species (ROS) levels, including H_2_O_2_. In resistant GBM cells, moderate H_2_O_2_ activates AKT, driving SH3GLB1 expression and sustaining drug resistance, whereas excessive H_2_O_2_ (e.g., following HNE co-treatment) suppresses SH3GLB1 and impairs resistance. Furthermore, HgCl_2_ downregulates mitochondrial aquaporin-9 (AQP9), reducing H_2_O_2_ flux and SH3GLB1 expression. This schematic illustrates how H_2_O_2_ levels, AKT activation, and AQP9 modulation collectively shape SH3GLB1-driven resistance

According to [38], endogenous H_2_O_2_ levels of approximately 0.001–0.1 μM are generally considered “physiological”. In the 0.05–1 μM range, often termed “eustress”, H_2_O_2_ can stimulate tumor cell proliferation. However, once concentrations rise to 1–10 μM, “oxidative distress” dominates and initiates cell death pathways. This gradient helps explain why varying H_2_O_2_ levels can differentially shape cell fate plasticity in GBM cells. Low-degree H_2_O_2_ can oxidize the cysteine residues of proteins to form a reversible sulfenic form (cysteine-SOH). However, higher oxidation results in irreversible cysteine-SO_2_H/SO_3_H formation, leading to loss of function [42]. Though more research is needed, we illustrated the potential weak point of resistant cells that could be utilized for drug development, such as pharmacologic ascorbic acid (≥20 mM) to induce high flux of H_2_O_2_ for combination with radiation/TMZ in GBM [36,43]. Recent reports indicate that high-dose vitamin C, investigated in various cancers (including breast and gastric), can generate a high flux of H_2_O_2_ and potentially enhance chemotherapy or radiotherapy outcomes [44–46]. Similarly, conventional agents such as cisplatin boost ROS levels to drive tumor cell death [46], while certain herbal compounds (e.g., cordycepin, evodiamine) exhibit cytotoxic effects by elevating ROS [47]. Although these approaches are not yet standard of care, they underscore the feasibility of leveraging increased ROS to eradicate cancer cells. Future research should establish the safety, optimal dosage, and timing of such pro-oxidant strategies when combined with TMZ to overcome therapy resistance in GBM. Collectively, the resistant cells possessed limited ROS balance regulation, and ROS fluctuations resulted in distinct SH3GLB1 expression, which affected TMZ susceptibility. In summary, SH3GLB1 was regulated via ROS-H_2_O_2_-AKT signaling (Fig. 6). Finally, we propose possible therapeutic strategies for future studies by increasing the ROS burden or influencing mitochondrial function to regulate SH3GLB1 levels to mediate tumor progression.

In fact, we used cell lines and derived resistant clones, which may not capture the full cellular diversity of patient tumors. We also did not examine our model in mitophagy or immune-competent *in vivo* systems, so interactions with damaged mitochondria clearance or the tumor microenvironment and host immunity remain to be defined. Our mechanistic focus was on hydrogen peroxide, and we did not dissect reactive nitrogen species (RNS). Nitric oxide and downstream species such as peroxynitrite can modulate signaling and therapy responses in GBM and other brain cancers [48], which suggests future work should further examine RNS-directed perturbations and effects.

## 5 Conclusion

In conclusion (Fig. 6), we show that the SH3GLB1-H_2_O_2_ pathway, controlled by the cell’s redox state, is a key driver of TMZ resistance in GBM. Moderate increases in H_2_O_2_ coincided with AKT activation and up-regulation of SH3GLB1 during TMZ treatment. In contrast, lowering H_2_O_2_ or driving it to high levels reduced SH3GLB1 and increased TMZ sensitivity. These results support combined strategies that adjust H_2_O_2_ handling and target SH3GLB1-related signaling and functions, such as AKT or protective autophagy, to overcome resistance.

## Data Availability

All data found in the present study are available in the article and as supplementary data.

## Abbreviations

AKT: Protein Kinase B
AQP9: Aquaporin9
ATZ: 3-Amino-1,2,4-triazole
CAT: Catalase
CCCP: Carbonyl cyanide m-chlorophenyl hydrazone
DETC: Sodium diethyldithiocarbamate trihydrate
DMEM: Dulbecco’s modified Eagle’s medium
FBS: Fetal bovine serum
GBM: Glioblastoma
GBM-R: Recurrent glioblastoma
GPx: Glutathione peroxidase
H_2_O_2_: Hydrogen peroxide
HNE: 4-hydroxynonenal
IHC: Immunohistochemistry
ΔΨm: Mitochondrial membrane potential
NAC: N-acetyl-L-cysteine
ROS: Reactive oxygen species
SH3GLB1: SH3 domain GRB2-like endophilin B1
SOD2: Superoxide dismutase 2
TCGA: The Cancer Genome Atlas
TMZ: Temozolomide
